# Inverse Bayesian inference in swarming behaviour of soldier crabs

**DOI:** 10.1098/rsta.2017.0370

**Published:** 2018-11-12

**Authors:** Yukio-Pegio Gunji, Hisashi Murakami, Takenori Tomaru, Vasileios Basios

**Affiliations:** 1 Department of Intermedia, Art and Science, School of Fundamental Science and Engineering, Waseda University, 3-4-1 Ohkubo, Shinjuku, Tokyo 169-0072, Japan; 2 Department of Information System Creation, Faculty of Technology, Kanagawa University, 3-27-1 Rokkakubashi, Kanagawa-ku, Yokohama-shi, Kanagawa 221-8686, Japan; 3 Interaction and Communication Desing Laboratory, Toyohashi University of Technology, 1-1 Hibarigaoka, Tempaku-cho, Toyohashi, Aichi 441-8580, Japan; 4 Department of Statistical Physics and Complex Systems, Université Libre de Bruxelles, Boulevard du Triomphe, 1050 Brussels, Belgium

**Keywords:** swarm, Bayesian inference, polarization, soldier crabs, collective behaviour

## Abstract

Animals making a group sometimes approach and sometimes avoid a dense area of group mates, and that reveals the ambiguity of density preference. Although the ambiguity is not expressed by a simple deterministic local rule, it seems to be implemented by probabilistic inference that is based on Bayesian and inverse Bayesian inference. In particular, the inverse Bayesian process refers to perpetual changing of hypotheses. We here analyse a time series of swarming soldier crabs and show that they are employed to Bayesian and inverse Bayesian inference. Comparing simulation results with data of the real swarm, we show that the interpretation of the movement of soldier crabs which can be based on the inference can lead to the identification of a drastic phase shift-like transition of gathering and dispersing.

This article is part of the theme issue ‘Dissipative structures in matter out of equilibrium: from chemistry, photonics and biology (part 2)’.

## 1. Introduction

As Ilya Prigogine proposed the notion of dissipative structure [1], biological structures as well as the dynamics of population and collective motion drew an extensive and intense interest for studies inspired by this approach. One of the early pioneers in uncovering the dynamical characteristics of collective motion was one of his first students, Jean-Louis Deneubourg. His seminal papers (see, for example, [2] and references within) viewed collective decision-making in ants as a bifurcation process. Subsequently, he and his co-workers opened a wide area of investigations uncovering the dynamical basis of self-organization in collective dynamics [3]. The influence of fluctuations and their complex interplay with structure and function, as it was first pointed out in [1], has ever-since been a far reaching frontier of the research in dynamical systems, including dynamical systems comprising many interacting elements, or ‘agents’. The term ‘agent’, as it has now been established in the literature, can be any entity which is endowed by specific rules of motion; it can mean a particle, a bacterium, a cell, an animal or even a robot [4,5].

Decision-making, in biology, the motion it induces and the resulting aggregation patterns brings together collective motion of animals (agents) and the dynamical interplay between individuals and groups. At the same time, it brings together nonlinear dynamics, statistical mechanics, probabilistic methods and the physics of complex systems under a new paradigmatic thinking. This approach to complex systems, to which this work also subscribes, was indeed inspired by the original ideas of Prigogine, Deneubourg and their co-workers. Phase-transitions, dissipative structures and their dynamical bifurcations underlying collective motion are now established as a widespread area of study. For a short statement article, one might refer to [6], where the multi- and inter-disciplinary fashion of the subject is highlighted.

While the swarm movements mentioned herein can be expressed as a nonlinear dynamical system coupled with perturbation, real swarm movements can be beyond a sole, specifically deterministic, rule because the swarm movement can rapidly and drastically change its behaviour. It looks as if the swarm movement sometimes follows a specific rule, but sometimes it does not follow the same rule at all. Therefore, it becomes evident and realistic that one has to extend the ideas coming from dissipative structures to such a dynamical system. We hope our work contributes to this direction, using real swarm data and ideas coming also from probability theory that provide a link with this kind of complex dynamics, especially their phase-like transitions.

Does a swarm, flock or school have one sociality and/or one unity? This question is being addressed by modelling collective decision-making using computer models [7–9], including BOIDS [10] and Self-Propelling Particle [11]. Recent developments in image analysis have made it possible to obtain kinetic data on how real organisms move [12,13], revealing internal dynamical structures within a group such as topological distance [14], scale-free correlation [15] and inherent noise [16]. It has also been suggested that inherent turbulence could play an essential role in collective motion [17–20]. Thus, the problem still remains on the relationship between sociality and inherent noise.

A swarm, flock and school can be typically explained by a computer model based on local neighbourhood interactions coupled with external noise [21–23]. In this sense, sociality implemented by local neighbourhood interaction could conflict with noise which can be compared to the freedom of individuals in a society. It seems to be difficult to coexist sociality with freedom because freedom or noise could come outside of the social rule.

We have previously shown that sociality and freedom of individuals could coexist in animal groups under the mechanism of mutual anticipation and asynchronous updating. We have also shown how inherent noise can actively contribute to the establishment and maintenance of a robust swarm acting as one unity by comparing a computer model based on an organism's mutual anticipation with kinetic data from soldier crabs [24–28] and fish schools [29]. Both in the model and real data, a swarm can be characterized as having high density and a wide variety of polarization. It shows coexistence of sociality and freedom. Mutual anticipation could explain temporal deviation of social rule and demonstrates that a swarm of soldier crabs can enter and cross the water while they never enter the water under normal conditions [18,20,21]. Both our model and kinetic data reveal the scale-free correlation of which correlation function is linearly decays independently on the swarm size [24,30]. It strongly suggests that a swarm acts as one body. The model should have wide applicability to biological collective phenomena in general.

The next question arises how mutual anticipation can be implemented in the perspective of animals. In our previous model, it is assumed that an animal can anticipate swarm mates' subsequent moves with each other. It suggests that an animal can infer the swarm mates’ subsequent moves and behaviours. One of the most hopeful candidates for the mechanism of anticipation is Bayesian inference [31–33]. Since future moves of other individuals cannot be observed from each animal, future moves can be estimated with probability [34,35]. The classical probability theory, however, contributes less efficiently to an animal's decision-making because possibilities of events are too large to compute probability. Bayesian inference can, however, reduce redundant possibilities which have nothing to do with actual experiences. That is why Bayesian inference is regarded as an intrinsic mechanism of cognition and/or perception not only for human brain [36–39] but also for various animals [40–46].

A shortcoming of Bayesian inference was recently pointed out [47,48]. In Bayesian inference, there is a set of hypotheses in advance, and the probability of the distribution of hypotheses is perpetually changed. The agent employed to Bayesian inference can make a decision by using the probability of hypotheses. Although the probability of hypotheses can be changed in Bayesian inference, a set of hypotheses cannot be changed. Since a set of hypotheses is prepared in advance and is fixed, the agent cannot deal with unexpected events which are not prepared in the hypotheses [49,50]. If another external stimulus invades the inference process in keeping on inference, a set of hypotheses could be influenced by the stimulus and could be modified qualitatively [47,51]. Therefore, due to the external and internal reason, a set of hypotheses is destined to be changed.

We recently introduced an idea in which a set of hypotheses is perpetually changed in Bayesian inference into the process of Bayesian inference, and call such a process inverse Bayesian inference [48–50,52]. Bayesian inference can be compared to an exploitation process, whereas the inverse Bayesian process can be compared to an exploration process. A pair of exploitation and exploration processes could contribute to an essential property of animal behaviour such as the Lévy walk [53]. A pair of Bayesian and inverse Bayesian (BIB) inference can contribute to animals' inference process under open environments.

In this paper, first we show how inverse Bayesian inference can contribute to Bayesian inference. Secondly, we show that our model animals, soldier crabs, could employ BIB inference. Finally, through simulating studies of the model based on BIB inference, we show that phase transition-like temporal change of polarization found in the swarm of soldier crabs could be explained by switching of approaching or avoiding a swarm resulting from BIB inference.

## 2. Bayesian and inverse Bayesian inference

Bayesian inference is now interpreted as an essential cognitive mechanism to an external stimulus. Evidence of Bayesian (B) inference is found not only in human cognition but also in various animals. An agent employing B-inference has multiple hypotheses and changes the distribution of probability of hypotheses dependent on empirical data. The probabilities of useless hypotheses are decreased dependent on the agent's experience, and those of hypotheses consistent with the agent's experience are increased and to be used for decision-making. If the environment is fixed, there can be one optimal solution. An agent employing B-inference can immediately reach the optimal solution. Because the natural environment, however, changes perpetually, an agent must prepare various hypotheses to cope with unknown conditions. Nevertheless, B-inference has only finite numbers of hypotheses which are invariant through inference. Inverse Bayesian inference is, therefore, added to B-inference to change the hypotheses dependent on empirical conditions.

First, we sketch B-inference in the following. The agent has a hypothesis set, {*h*_0_, *h*_1_, …, *h_m_*_−1_} and identifies the state of the environment as data in a dataset, {*d*_0_, *d*_1_, … , *d_n_*_−1_}. The probability of hypothesis, *h*, data, *d*, and conditional probability of hypothesis, *h*, under given data, *d*, are expressed as *P*(*h*), *P*(*d*) and *P*(*h*|*d*), respectively. Each hypothesis, *h*, is defined by the likelihood of which the distribution of the probability of data is determined, such as *P*(*d*|*h*), the conditional probability, *d*, of data under the hypothesis, *h*. Because of the definition of conditional probability, *P*(*h*|*d*) = *P*(*h, d*)/*P*(*d*) and *P*(*d*|*h*) = *P*(*d, h*)/*P*(*h*), and *P*(*h, d*) = *P*(*d, h*), one can obtain *P*(*h*|*d*)*P*(*d*) = *P*(*d*|*h*)*P*(*h*). As *P*(*d*) = Σ*_k_P*^*t*^(*d*|*h_k_*),
2.1
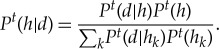

The superscript of *t* in *P*^*t*^(*h_k_*) represents the probability of *h_k_* at *t*th step, because the probability is changed step-wisely. In B-inference, the probability of hypothesis under a given data, *P*(*h*|*d*), is interpreted as ‘*a posteriori* probability’ of hypothesis, where the probability of hypothesis before obtaining data, *P*(*d*), is interpreted as ‘*a priori* probability’ of the hypothesis. Iterative inference makes preceding *a posteriori* probability subsequent to *a priori* probability. To distinguish ‘preceding’ from ‘subsequent’ probability in the iterative inference, the probability of the hypothesis at *t*th step is represented by *P*^*t*^(*h*). Thus, replacing subsequent *a priori* probability of the hypothesis with preceding *a posteriori* probability is expressed as follows:
2.2
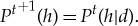


If the likelihood of hypothesis, *P*(*d*|*h*), is invariant, i.e. *P*^*t*^^+1^(*d*|*h*) =* P*^*t*^(*d*|*h*), any hypothesis is not changed and only distribution of the probability is changed dependent on data. By contrast, inverse Bayesian inference (IB inference) changes the likelihood of a specifically chosen hypothesis, *h*_s_, in the form of
2.3
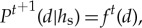

where *f*^*t*^(*d*) represents a normalized frequency of the occurrence of data, *d*, at the time interval, *M*. Let a dataset {*d*_1,_ *d*_2_, *d*_3_} and *M *= 4. Given a time series of data at *t* − 5, *t* − 4, … , *t* as *d*_0_, *d*_1_, *d*_1_, *d*_2_, *d*_3_, *f*^*t*^(*d*_0_) = 0.0, *f*^*t*^(*d*_1_) = 0.5, *f*^*t*^(*d*_2_) = 0.25 and *f*^*t*^(*d*_3_) = 0.25. Similarly, *f*^*t*^^−1^(*d*_0_) = 0.25, *f*^*t*^(*d*_1_) = 0.5, *f*^*t*^(*d*_2_) = 0.25 and *f*^*t*^(*d*_3_) = 0.0. A specific hypothesis, *h*_s_, is chosen as the least optimal hypothesis (i.e. for any *h* ∈ {*h*_0_, *h*_1_, … , *h_m_*}, *P*^*t*^(*h*_s_) ≤* P*^*t*^(*h*)) with the probability of 1 − *P*^*t*^(*h*_s_). As *P*^*t*^(*h*_s_) ≤* P*^*t*^(*h*), *h*_s_ can be chosen as the least optimal hypothesis with the highest probability. As *f*^*t*^(*d*) represents the temporal probability of data, like *P*^*t*^(*d*), it is easy to see that equation (2.3) is symmetric to equation (2.2). That is why the inference equipped with equation (2.3) is called IB inference. If an agent employs both B-inference and IB inference, the system is called the BIB inference system.

**Figure 1.**
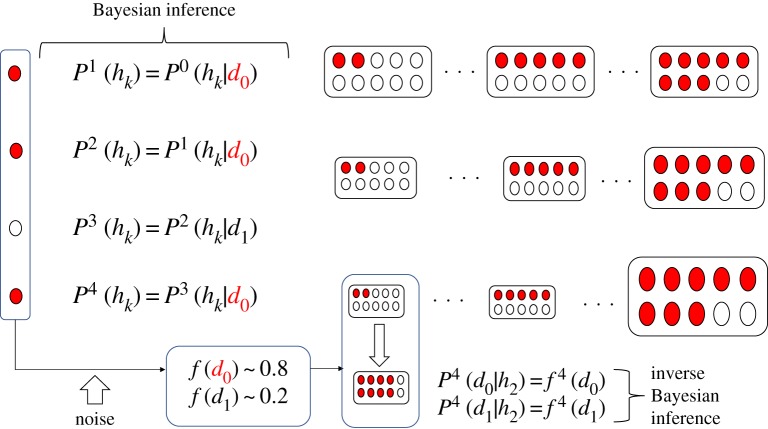
Schematic diagram of Bayesian and inverse Bayesian inference. In this system, a set of data consists of white and red balls, and a hypothesis is expressed as a bag containing 10 balls which are red or white. Time proceeds downwardly, and a set of hypotheses is represented by a collection of bags at each row. The size of a bag represents the probability of a hypothesis, which is temporally changed dependent on experience, resulting from Bayesian inference. The frequency of occurrence of data could substitute into the likelihood of a specific hypothesis, which is inverse Bayesian inference. (Online version in colour.)

Figure 1 shows a schematic diagram of BIB inference. Given a bag containing 10 balls consisting of either red or white balls, one ball is taken from the bag, is identified as either red or white and is returned to the bag. This process is repeated. The agent is asked to infer the content of a bag, where he has a finite number of hypotheses (bags, drawn as a diagram). A set of data is defined by {*d*_0_, *d*_1_} and *d*_0_ (resp. *d*_1_) represents red balls (resp. white balls), and a set of hypotheses is defined by a possible bag containing balls. Time proceeds from top to bottom. Given the data at each time step, the agent replaces the probability of hypotheses with the conditional probability of hypothesis under the given data, which is B-inference. This leads to the change of the distribution of the probability of hypothesis. In figure 1, the size of the bag represents the probability of hypothesis. At the fourth step, the time series of data, *d*_0_, *d*_0_, *d*_1_, *d*_0_, constitutes *f*(*d*_0_) ∼ 3/4 and *f*(*d*_1_) ∼ 1/4. These probabilities of data are substituted for the likelihood of the least optimal hypothesis, *h*_2_, which is IB inference.

**Figure 2.**
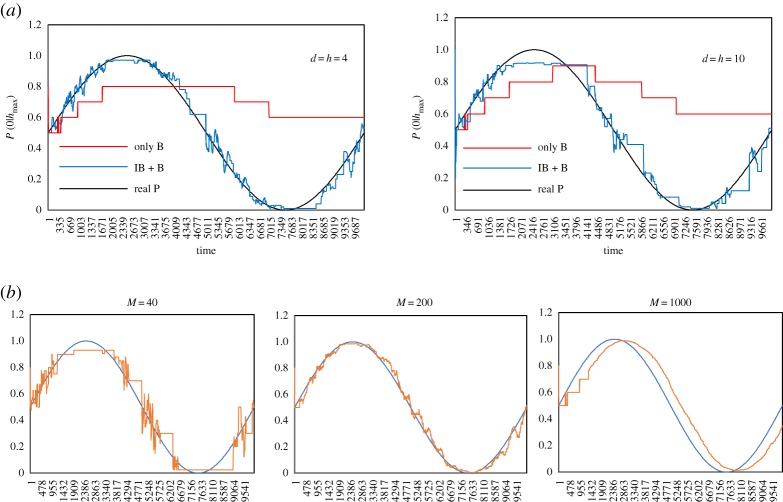
Comparison of decision-making based only on Bayesian inference and that on Bayesian and inverse Bayesian inference (BIB inference) with a given time series of the probability of data controlled by the sin-curve. The number of data and hypotheses are represented by the number (*a*). Comparison of decision-making based on BIB inference (orange lines) with a given time series of the probability of data (blue lines) where the interval of the frequency (*M*) is changed for each graph (*b*). (Online version in colour.)

Figure 2*a* shows how IB inference contributes to the inference. Under this condition, the number of data is the same as the number of hypotheses and is 4 (*d*_0_, *d*_1_, *d*_2_, *d*_3_) or 20 (*d*_0_, … , *d*_19_). The probability of *d*_0_, *P*(*d*_0_), is temporally changed along the sin-curve, and is represented by black lines. Decision-making of an agent is estimated by *P*(*d*_0_|*h*_max_), where *h*_max_ represents the optimal hypothesis such that *P*(*h*_max_) > *P*(*h*) for any hypothesis *h*. Red lines represent *P*(*d*_0_|*h*_max_) obtained only by B-inference, and blue lines represent *P*(*d*_0_|*h*_max_) obtained by BIB inference. Only B-inference cannot follow the drastic change of *P*(*d*_0_) and traces the cumulative frequency of *P*(*d*_0_). By contrast, BIB inference can immediately trace the change of *P*(*d*_0_), which is controlled by the sin-curve. Because the likelihood of a chosen hypothesis is perpetually replaced by the normalized frequency of data, the new hypothesis can contribute to decision-making so far as the probability of data is temporally changed. Under this simulation *M* for the normalized frequency is set as 100.

Figure 2*b* shows how the value of *M* can influence the error between *P*(*d*_0_) controlled by the sin-curve and *P*(*d*_0_|*h*_max_) obtained by BIB inference. If *M* is too small (e.g. *M *= 40), normalized frequency of data, *f*(*d*), is too sensitive to estimate the stochastic change of *P*(*d*_0_). Thus, the probability of data controlled by the sin-curve can be traced with large error. By contrast, if *M* is too large, *f*(*d*) cannot reflect the change of *P*(*d*_0_). Thus, *P*(*d*_0_|*h*_max_) consistent with *P*(*d*_0_) is always delayed. This results in perpetual large amounts of error between *P*(*d*_0_) and *P*(*d*_0_|*h*_max_). In a broad region of *M*, the small error can be achieved between *M *= 50 and 300. As a result, we can choose optimal *M* in a broad region.

As BIB inference can follow the drastic change of data, i.e. the temporal change of environment, it suggests that animals seem to infer the environment using not only the B-inference but also the IB inference system. In the next section, we adopt a model animal, soldier crabs, *Mictyrus guinotae*, and show that they could use BIB inference.

## 3. Bayesian and inverse Bayesian inference in a swarm of *Mictyrus guinotae*

### (a) Material and methods

We use soldier crabs, *M. guinotae*, as a model organism to compare kinetic data on swarming with the computer model because they live in lagoons and form a huge swarm composed of several hundred to hundreds of thousands of individuals. Individuals for the experiments were collected from a large colony of *M. guinotae* at Funaura Bay on Iriomote Island, Okinawa Prefecture, Japan (123°48′ E; 24°24′ N) during the day from 1.5 h before to 1.5 h after low tide in September 2016. Individuals (10, 20, 40 and 100 individuals at each trial) were released in the experiment arena of (0.8 × 1.4 × 0.2) m, in which the substrate was covered by muddy sand collected from Funaura Bay of 1 cm depth. The wall of the arena was covered by flat black tape, which appeared to relax the crabs. The experiment was conducted at Iriomote Station, Tropical Biosphere Research Center, University of the Ryukyu. Soldier crabs were left in the arena for 2 h at 28°C. All experiments were recorded from above using a Panasonic HDC-TM700 camcorder (1920 × 1080 pixels, 30 frames s^−1^) fitted with a Panasonic VW-W4907H-K wide conversion lens (0.75×) on a steel frame, resulting in a recording area of 1 × 1.7 m.

From the obtained greyscale images, time series of identified individuals' positions were tracked by using image-processing software (Library Move-tr/2D v. 8.31; Library Co. Ltd, Tokyo, Japan) in which each crab's positions were identified by the white paper-made marker attached to the crab's back, which appeared lighter than the surrounding area; the crab trajectories were constructed by tracking individuals from one frame to the next. When crabs overlapped or were in contact with others, we separated them using the manual tracking mode of the software. As a result, we obtained all individuals' *x* – *y* coordinates as a single pixel whose side length was 4.76 mm, for each observed time duration. In this study, the time interval between two consecutive reconstructions of individuals’ coordinates was *dt *= 0.1 s (12 frames).

### (b) Swarming behaviour in experimental condition

**Figure 3.**
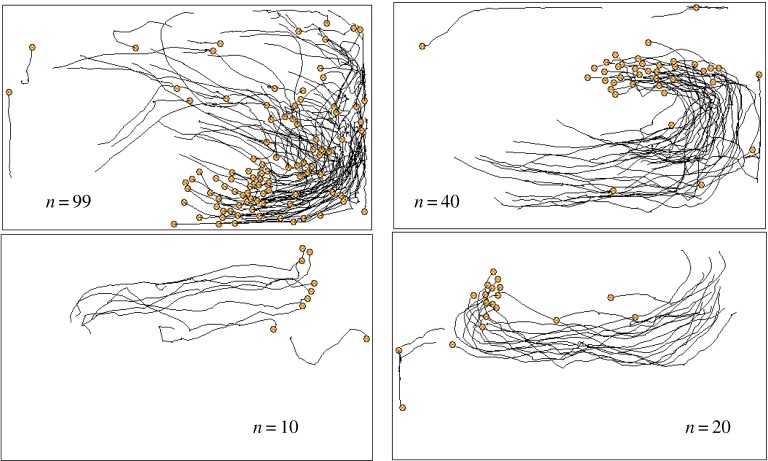
Snapshots of the real soldier crabs, *Mictyris guinotae*, wandering in a tank under the laboratory condition. An individual is represented by a circle accompanied by its previous trajectory. (Online version in colour.)

Figure 3 shows snapshots of trajectories for each population, where the number represents the population size. Orange circles represent the position of each crab at a certain time, and the black curves accompanying the orange circles represent the trajectories to reach the current position within 60 s. When soldier crabs were kept in a plastic container under light experimental conditions, they randomly and rapidly moved, tried to escape from the container independent of other individuals and there was no swarming behaviour. Soldier crabs in our experimental arena, however, were calmed down, moved slowly and showed schooling behaviour. They moved in alignment with other individuals not only along the wall but also in the central region of the arena. Schooling behaviour was easily observed independent of population size. While most individuals gathered together, and constituted a dense swarm, delayed crabs followed the swarm. There were some exceptions that some crabs avoided the swarm and moved independent of the swarm. The character of the avoiding swarm never resulted from the character of the individual, because crabs which previously avoided the swarm could be attracted by the swarm and vice versa. It suggests that an individual soldier crab determines whether it goes toward or avoids the swarm dependent on its own experience.

**Figure 4.**
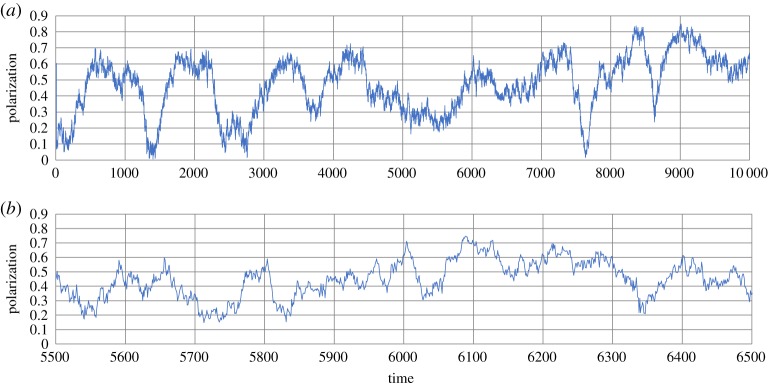
Polarization of the real soldier crabs, *Mictyris guinotae*, plotted against time. Hundred individuals (*a*) and 40 individuals (*b*). (Online version in colour.)

Figure 4*a* shows polarization of the swarm against time, where the population consists of 100 individuals. Polarization, *ψ*, is defined by
3.1
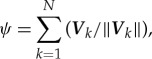

where ***V****_k_* represents a velocity vector of the *k*th individual, and ‖***V***‖ represents the norm of the vector. The population consisting of 100 individuals changed polarization drastically. This reveals that a swarm-satisfying alignment was autonomously collapsed, dispersed and then dispersed individuals gathered together to constitute a swarm. An individual sometimes was attracted to, and sometimes avoided a dense area of individuals. The timing of gathering and dispersing seems to be synchronized, resulting from collective behaviour. It suggests that an individual could anticipate another individual's moves and mimic neighbours’ behaviour with each other. Figure 4*b* also shows polarization of the population of soldier crabs plotted against time, where the population consists of 40 individuals. This small population also shows temporal changing of the polarization, while the duration in which an alignment of swarm maintains is shorter than the duration of alignment in a bigger swarm. As well as the population consisting of 100 or 40 individuals, any other smaller populations (*N *= 10, 20) show a drastic change of the polarity against time.

If the swarming behaviour is based on alignment rule and/or flock centring, once the swarm is generated, then the swarm moves with perfect alignment and cannot be collapsed. Although perturbation for the alignment rule can play a role in alternation of gathering and collapse, such perturbation can make the boundary of a dense swarm vague. It results in vague gathering and vague dispersing, and never produces a drastic change in the collapse of alignment and sudden gathering as shown in figure 4. Therefore, the clear alternation of gathering and dispersing, and/or drastic change of polarization against time, could suggest individual anticipation and adjustment of moving under asynchronous updating. That is the only way to coexistence of gathering and dispersing. Reciprocal anticipation can give rise to synchronized gathering; however, it can amplify errors in anticipation and then give rise to synchronized dispersing because of asynchronous updating.

The next question arises, how an individual could anticipate other neighbours’ behaviours with each other. It can be based on BIB inference. In the next section, we estimate whether soldier crabs are employed to BIB inference or not.

### (c) Approaching or avoiding swarm based on Bayesian and inverse Bayesian inference

We here assume that individuals of soldier crab are employed to the following decision-making system based on BIB inference (figure 4). Coordinate of the *i*th individual at *t*th time step is expressed as 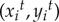. A set of data, *D* = {0, 1, 2, 3}, where each data represent relative density in the neighbourhood. Let 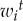 the number of individuals at *t*th step in neighbourhood of *i*th individual at *t*th step with radii, *r*, and 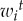 that with radii, 2*r*, where the centre of the neighbourhood is the same as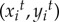. The data, *d* ∈ *D*, which represent the *i*th individual's state at *t*th step, are expressed as follows:
3.2
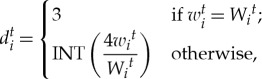

where for real number *x*, INT(*x*) is an integer part of *x*.

A set of hypothesis, *H* = {*η*_0_, *η*_1_, *η*_2_, *η*_3_}, is defined by the likelihood in the form of *P*(*d*|*h*). If an agent is employed to only Bayesian inference, the likelihood of a hypothesis is time invariant. In BIB inference, the likelihood is temporally changed. Thus, we define the likelihood of *η_k_* by
3.3
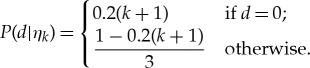


In only Bayesian inference, *η*_k_ defined by the above equation is used for any individual through time; however, in BIB inference, *η_k_* would be used only for initial conditions. Therefore, the likelihood of hypothesis is determined per individual at each time step.

We assume that individual soldier crabs obtain the data as equation (3.2), and that dependent on these data, it calculates conditional probability of hypothesis by
3.4
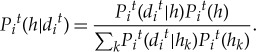

Then, by B-inference, conditional probability of hypothesis under *d_i_*^*t*^ is substituted into the probability of hypothesis itself by
3.5
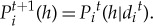


After that IB inference changes the likelihood of a hypothesis *h*_s_ in the form of
3.6
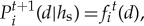

where 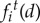 represents normalized frequency of *d* observed for *i*th individual in the interval between *t* and *t* − *M* step. It is assumed that by using 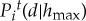, a soldier crab determines whether they approach or avoid a dense area of swarm mates where *h*_max_ satisfies 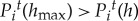 for any hypotheses *h*.

To estimate the efficiency of BIB inference in a swarm of soldier crabs, we compare 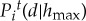 with 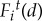 that is a normalized frequency of *d* in a subsequent time interval between *t* − *M*/2 and *t *+ *M*. We call 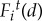 the probability of *d* in future. If the difference between 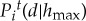 and 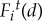 is small, it suggests that an individual would use 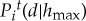 and data *d* can appear in a subsequent time.

**Figure 5.**
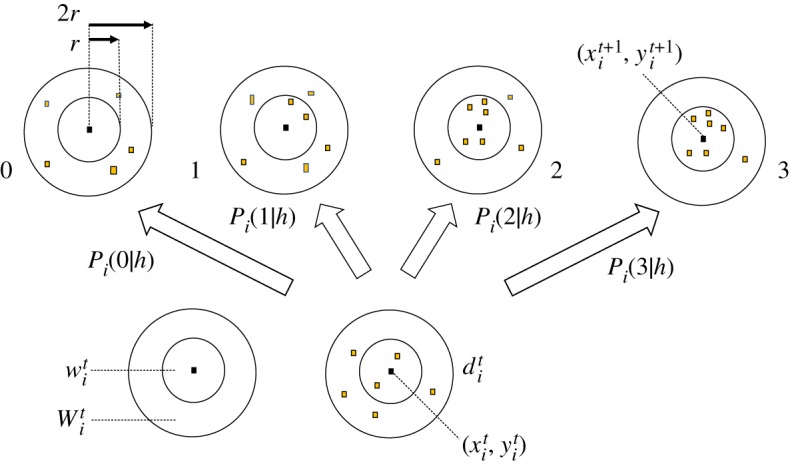
Schematic diagram of data and hypothesis adopted by a time series of real soldier crabs. (Online version in colour.)

**Figure 6.**
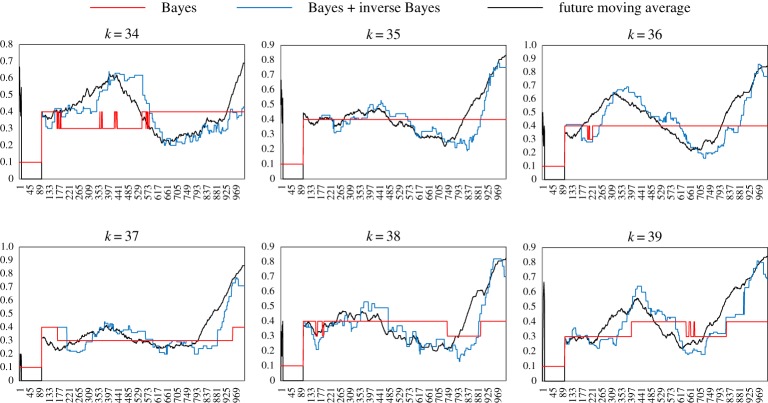
Comparison of decision-making (*P*(0|*h*_max_)) based only on Bayesian inference and that on BIB inference with a given time series of the probability of data in the subsequent time series, for an individual.

Figures 5 and 6 show comparison of 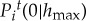 obtained only by B-inference and by BIB inference with 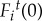 for some individuals. It is easy to see that decision-making based only on B-inference cannot follow perpetual change of 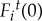, and that decision-making based on BIB inference can be consistent with 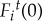. As 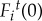 is temporally changed from very high to very low, it shows that individuals sometimes avoided swarm mates (high 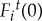) and sometimes approached a dense part of the swarm (low 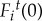). As 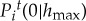 obtained by the BIB inference is always correlated with 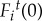, it suggests that solder crabs use not only Bayesian but also inverse Bayesian inference to make a decision to move.

**Figure 7.**
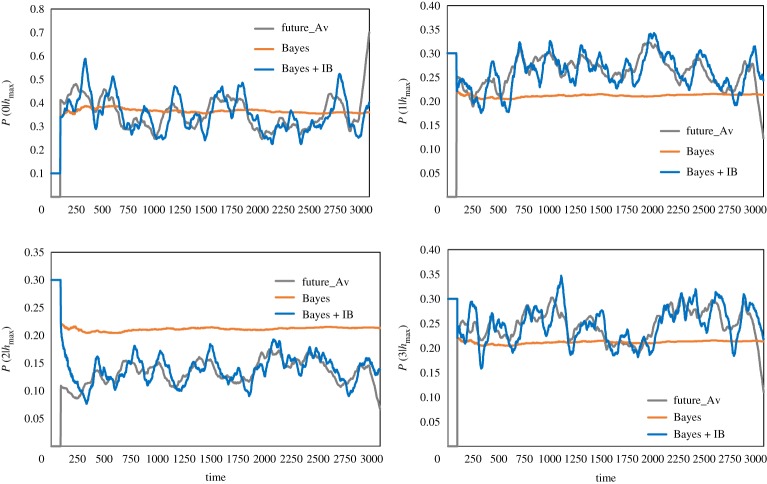
Comparison of decision-making (*P*(0|*h*_max_)) based only on Bayesian inference and that on BIB inference with a given time series of the probability of data in the subsequent time series, in terms of the average of the population.

**Figure 8.**
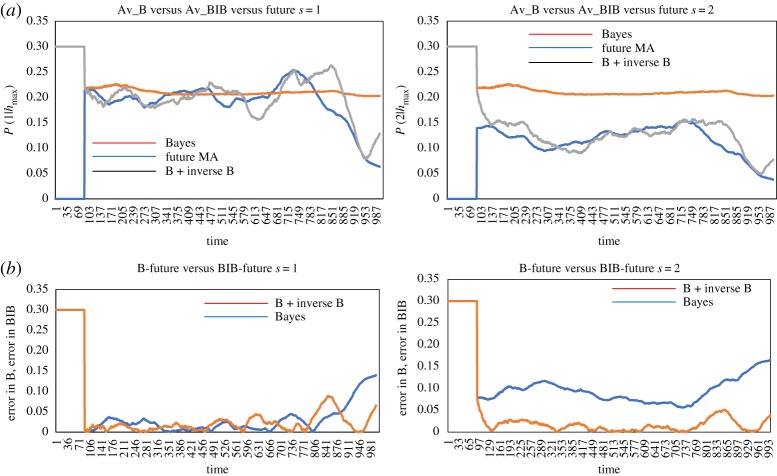
Comparison of decision-making (*P*(0|*h*_max_)) based only on Bayesian inference and that on BIB inference with a given time series of the probability of data in the subsequent time series, in terms of the average of the population.

Figure 7 shows comparison of 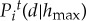 obtained only by B-inference and by BIB inference with 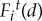 with respect to the average of all individuals in a population consisting of 99 individuals. Each graph shows the conditional probability of the data, 0, 1, 2 or 3. While 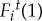 and 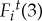 moved almost at 0.25, which is expected by random probability, 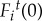 is higher than and 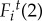 is lower than the random average. It implies that an individual often moved to homogeneously dense parts in the swarm and avoided the dense part. That is why the dense swarm appeared, was kept for a while and then members of the swarm were dispersed. This process was repeated, and that could give rise to dynamic changing of polarization as shown in figure 4. Figure 8 shows another time sample of a time series of the population consisting of 100 individuals. Figure 8*a* shows *P*^*t*^(*d*|*h*_max_) (*d *= 1, left and *d *= 2 right) based only on B-inference, BIB inference and *F*^*t*^(*d*) for the average of all individuals plotted against time. Especially for *P*^*t*^(2|*h*_max_), it is easy to see that decision-making based on BIB inference is much more consistent with *F*^*t*^(*d*). It shows that a soldier crab could use BIB inference for moving in a swarm.

The average of making decisions based only on B-inference, however, did not reveal dynamic changing 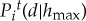. Thus, it cannot explain dynamic polarization of the swarm. In the next section, we propose a swarm model based on BIB inference and evaluate whether the swarm model can lead to dynamic changing of polarization of the swarm.

### (d) The swarm model implemented by Bayesian and inverse Bayesian inference

**Figure 9.**
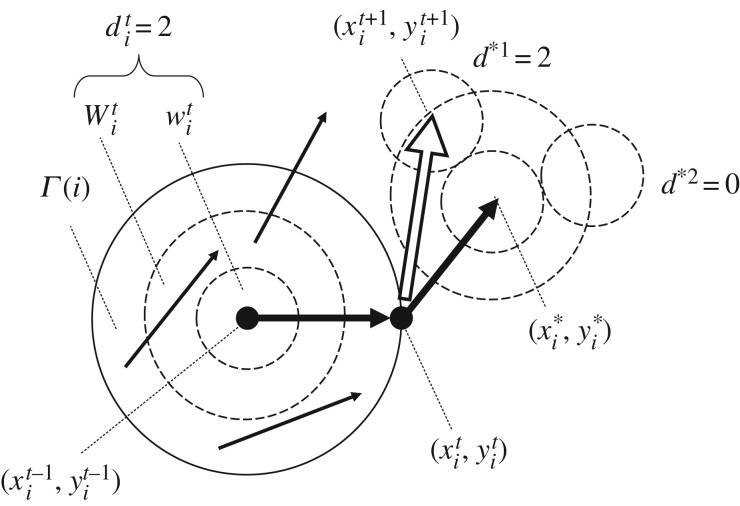
Schematic diagram for the swarm model based on BIB inference. Anticipated velocity, which is 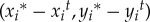, results from averaging of the velocities of neighbours (in *Γ*(*i*)). Simultaneously, data for *i*th individual at *t*th step, 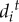 is obtained in a broken circular neighbourhood in *W_i_*^*t*^. If the position around 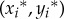 which is as same as 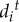 is found, the *i*th individual moves to that position at *t *+ 1th step.

We here modify the swarm model implementing the alignment rule by adding BIB inference, as shown in figure 9. When the coordinate of an individual is expressed as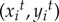, 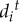 in *D* = {0, 1, 2, 3} is obtained by equation (3.2). Hypothesis, *h* in *H* = {*η*_0_, *η*_1_, *η*_2_, *η*_3_}, is initially (or in only B-inference) defined by
3.7
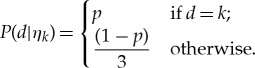

From 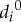, *a posteriori* probability of *h*, any hypothesis, is obtained by
3.8
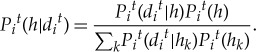

Owing to B-inference, 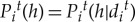 is obtained for any *h*, and then *h*_max_ such that 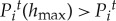 (*h*) for any *h*. Thus, the optimal data, *d*_max_, for *i*th individual at *t*th step is obtained such that for any *d*
3.9
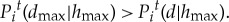

The anticipated coordinate, 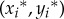, is obtained via alignment (figure 9). The alignment rule is applied to the velocity of an individual such that
3.10
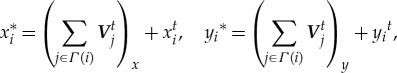

where 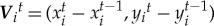, *Γ*(*i*) represents the circular neighbourhood with radii, *R*, whose centre is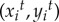, and (***V***)*_x_* represents *x-*component of vector ***V***. Around 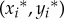 candidates of the next position are calculated such that
3.11
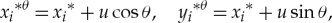

for discrete *θ* = 0, *π*/36, 2*π*/36, …, 2*π*, where *u* is a norm of the unit vector. For each 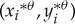, 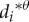 is calculated. The most optimal position is obtained such that
3.12
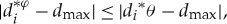

for any *θ* = 0, *π*/36, 2*π*/36, …, 2*π*. If there are some *φ* satisfying (3.12), one of them is randomly chosen and the next position of *i*th individual is determined as follows:
3.13
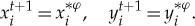


After the transition of the position, the likelihood of a hypothesis, *h*_s_, in the form of
3.14
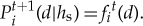

All control parameters of this model are *r*, the radii of the neighbourhood for calculating density (data); *p*, the probability of *P*(*d*|*η*_d_) employed to initial condition or only B-inference; *R*, the radii of the neighbourhood for alignment; *u*, the norm of unit velocity and *N*, the number of individuals.

**Figure 10.**
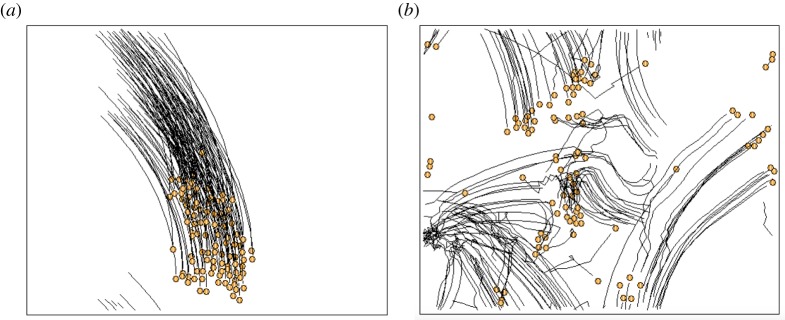
Snapshots of the swarm model based on BIB inference. Swarming phase (*a*) and dispersing phase (*b*). (Online version in colour.)

Figure 10 shows some snapshots of the simulating results of the swarm model based on BIB inference, where *r *= *R *= 20, *p *= 0.7, *u *= 1 and *N *= 100. Figure 10*a* shows a swarming phase in which polarization is very high and all individuals are aligned together. Figure 10*b* shows a dispersing phase in which polarization suddenly decreases by collapse of the swarm. In BIB inference, perpetual alternation of two phases of swarming and dispersing could occur due to the perpetual switching of approaching and avoiding dense parts of individuals resulting from inverse Bayesian inference. By contrast, the population employed only to B-inference cannot show alternation of two phases. If *p* is large enough to stable data, each hypothesis can inhibit switching between different data (i.e. switching between approaching and avoiding a dense part). It implies that once a swarm is generated, it cannot be collapsed. By contrast, if *p* is small enough not to stabilize specific data (e.g. *p* ∼ 0.25), the hypothesis continuously changes avoiding and approaching a dense part of the individuals, and then rigid dense swarm cannot be generated. Thus, the population employed only to B-inference, population shows either swarming with high polarization or dispersing population with low polarization. The population employing BIB inference can show phases shift between swarming and dispersing even if *p* is large enough.

**Figure 11.**
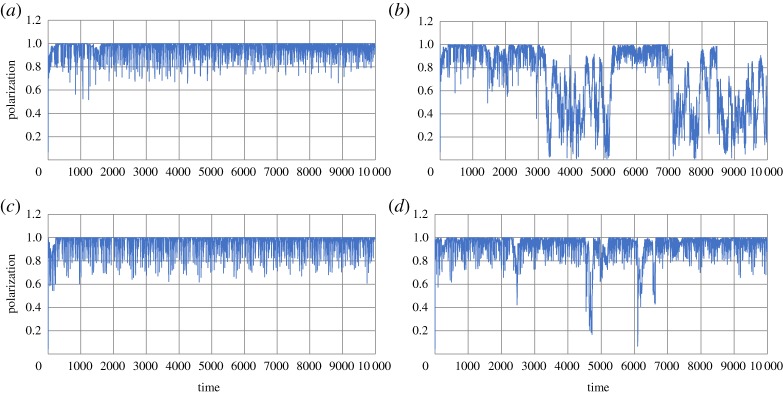
Polarization of the population employed only to B-inference plotted against time, where *p *= 0.7, *u *= 1, *r *= *R *= 30 (*a,b*), *r *= *R *= 50 (*c,d*) and *N *= 100 (*a,c*). Polarization of the population employed only to BIB inference plotted against time (*b,d*). (Online version in colour.)

Figure 11*a,c* shows polarization of the population employed only to B-inference plotted against time, where *p *= 0.7, *u *= 1, *r *= *R *= 30 (*a,b*), *r *= *R *= 50 (*c,d*) and *N *= 100. Soon after the initial condition, the swarm with high polarization is generated, it cannot be collapsed. Figure 11*b,d* shows polarization of the population employed only to BIB inference plotted against time, where the parameters are the same as the population employed only to B-inference. Even if the *p* is large enough to stabilize swarming, the effect of inverse Bayesian inference can introduce the possibility of dispersing and implement a newly generated hypothesis to collapse swarm. Therefore, even rigid and stable swarm is collapsed, while stable swarm is regenerated because inverse Bayesian inference can implement newly generated hypothesis to stabilize swarm.

**Figure 12.**
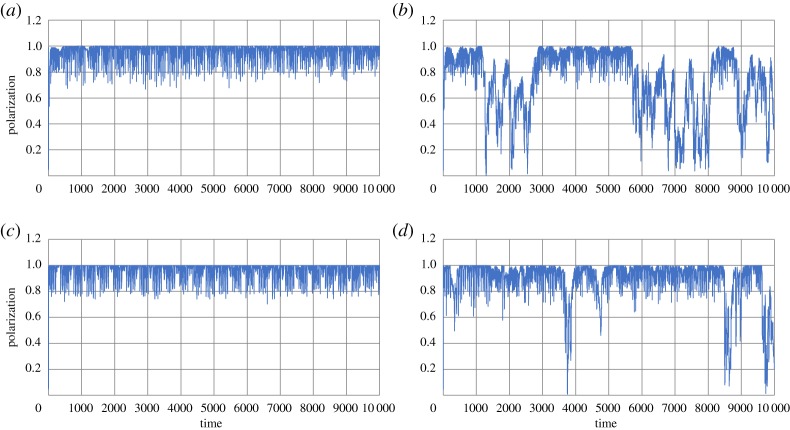
Polarization of the population employed only to B-inference plotted against time, where *p *= 0.99, *u *= 1, *r *= *R *= 20 (*a,b*), *r *= *R *= 40 (*c,d*) and *N *= 100 (*a,c*). Polarization of the population employed only to BIB inference plotted against time (*b,d*). (Online version in colour.)

Figure 12*a,c* shows polarization of the population employed only to B-inference plotted against time, where *p *= 0.99, *u *= 1, *r *= *R *= 20 (*a,b*), *r *= *R *= 40 (*c,d*) and *N *= 100. Figure 12*b,d* shows polarization of the population employed only to BIB inference plotted against time, where the parameters are the same as the population employed only to B-inference in figure 12*b,d*. It is easy to see that even if *p* is extremely high, the effect of inverse Bayesian inference can collapse and regenerate rigid and stable swarm. Such behaviours are found in natural swarms of soldier crabs as shown in figures 3 and 4.

From these results, we conclude that soldier crabs use not only Bayesian but also inverse Bayesian inference which can lead to switching between approaching and avoiding a dense part of individuals, and that the switching can give rise to perpetual alternation of swarming phase and dispersing phase.

## 4. Conclusion

As mentioned at first, freedom in a society might conflict the norm of society itself. Such a conflict might be artefact because the norm of society could be implemented as a deterministic rule. Even in the swarm model, if the norm of swarm is expressed as an alignment rule, freedom in a swarm is expressed as autonomously generated perturbation that can be implemented by random generator. By contrast, we investigated coexistence of freedom and norm of society under the assumption of mutual anticipation and asynchronous updating. If the time slice is assumed, then mutual anticipation and asynchronous updating can be implemented by inference equipped with probability. This can be expressed as a pair of BIB inference.

By Bayesian inference, an individual in a swarm can arrange the distance among individuals in moving and generate dynamic dense swarm, while by inverse Bayesian inference an individual can introduce possible dispersing and regenerating swarm and generate alternation of swarming and dispersing. Thus, not only Bayesian inference but also inverse Bayesian inference can contribute to dynamic swarming of animals. That is BIB inference.

In this paper, we show the swarming of soldier crabs, *M. guinotae*, and analyse the swarming behaviour with respect to polarization and performance of BIB inference. Polarization of soldier crabs is altered between swarming phase and dispersing phase. The performance of BIB inference is much more consistent with the actual probability of soldier crabs rather than only Bayesian inference. It shows that BIB inference can give rise to an individual's switching between approaching and avoiding a dense part of individuals, and then entails alternation of swarming with high polarization and dispersing with low polarization.

Dynamic coexistence of freedom and sociality in the animal group could be achieved by probabilistic inference opened to the external environment, which can be implemented by BIB inference.

## Data accessibility

This article has no additional data.
